# Developing Tobacco Control Interventions in Permanent Supportive Housing for Formerly Homeless Adults

**DOI:** 10.1177/1524839919839358

**Published:** 2019-04-11

**Authors:** Natalie M. Alizaga, Tram Nguyen, Anne Berit Petersen, Holly Elser, Maya Vijayaraghavan

**Affiliations:** 1 Cañada College, Redwood City, CA, USA; 2 University of California, San Francisco, CA, USA; 3 Loma Linda University, Loma Linda, CA, USA; 4 University of California, Berkeley, CA, USA; 5 Stanford University, Stanford, CA, USA

**Keywords:** permanent supportive housing, homeless adults, tobacco control, smoke-free policies

## Abstract

Smoke-free policies are effective population-based strategies to reduce tobacco use yet are uncommon in permanent supportive housing (PSH) for formerly homeless individuals who have high rates of smoking. In this study, we partnered with six supportive housing agencies in the San Francisco Bay Area to examine the implementation of smoke-free policies and cessation services. We administered a questionnaire and conducted in-depth, semistructured interviews with agency directors (n = 6), property management staff (n = 23), and services staff (n = 24) from 23 PSH sites on the barriers to implementing tobacco control interventions. All properties restricted smoking in indoor shared areas, but only two had policies restricting smoking in living areas. While there was staff consensus that smoke-free policies were important to reduce tobaccorelated harm, participants disagreed on whether smoke-free policies were aligned with PSH’s harm reduction framework. Residents’ comorbid mental illness and substance use and the lack of appropriate enforcement tools were barriers to implementation. Using these formative findings, we present a framework for a toolkit of strategies to increase implementation of smoke-free policies and cessation interventions in PSH. Successful implementation of indoor smoke-free policies in PSH will require concurrent cessation services to support smoking cessation efforts and address the mental health and substance use needs of residents.

**T**here are substantial cancer and cardiovascular disease disparities in the health of homeless adults compared with the general population (Baggett, Rigotti, & Campbell, 2016). Tobacco use largely contributes to these disparities (Baggett, LebrunHarris, & Rigotti, 2013). Although smoking prevalence in the United States is 15% (Jamal, King, Neff, Whitmill, Babb, & Graffunder, 2016), the prevalence among populations experiencing homelessness is estimated to be 70% to 80% (Baggett et al., 2013). Smoking-attributable cancer and cardiovascular disease are the leading causes of death among homeless individuals aged 50 years and older (Baggett et al., 2015), and heart disease and cancer are 3 times more common in homeless persons younger than 45 years compared to their agematched counterparts (Hwang, Wilkins, Tjepkema, O’Campo, & Dunn, 2009).

Smoke-free policies are effective population-based strategies to reduce tobacco use (International Agency for Research on Cancer, 2009). Living in a smoke-free home has been associated with decreased cigarette consumption and successful quitting among smokers and decreased exposure to secondhand smoke among nonsmokers (International Agency for Research on Cancer, 2009). Despite the benefits, low-income individuals are less likely to adopt smoke-free homes (Vijayaraghavan, Messer, White, & Pierce, 2013).

Permanent supportive housing (PSH) for formerly homeless adults may be an ideal environment to engage in smoking cessation behaviors because it offers a stable living environment (Petersen, Stewart, Walters, & Vijayaraghavan, 2018). PSH offers subsidized permanent housing with on-site or closely linked supportive services to formerly homeless adults, the vast majority of whom have co-occurring mental health and/or substance use disorders (Tsemberis, Gulcur, & Nakae, 2004). PSH utilizes a harm reduction framework, prioritizing housing stability without preconditions of abstinence (Tsemberis et al., 2004). Residents are required to pay 30% of their income on rent if they have an income and are encouraged to engage in supportive services. Studies have demonstrated PSH’s efficacy in increasing housing stability (Martinez & Burt, 2006), reducing health care utilization (Sadowski, Kee, VanderWeele, & Buchanan, 2009), improving health outcomes (Buchanan, Kee, Sadowski, & Garcia, 2009), and reducing adverse substance use–related outcomes (Collins et al., 2012).

Although smoke-free policies could increase quitting when combined with cessation services (Gilpin, Messer, & Pierce, 2006), a minority of PSH have such policies (Petersen et al., 2018). Results from a case study of one of the first PSH sites to have implemented smoke-free policies suggest that these policies are feasible (Petersen et al., 2018). Homeless adults are supportive of smoke-free policies in shelters, and many report that policies could facilitate smoking reduction (Arangua, McCarthy, Moskowitz, Gelberg, & Kuo, 2007; Businelle et al., 2015; Vijayaraghavan & Pierce, 2015b). However, shelter staff report several barriers to policy implementation, including the possibility of increasing unsheltered homeless (Businelle et al., 2015). Given the paucity of evidence on tobacco control interventions in PSH, additional research is needed to understand potential barriers to and facilitators of implementing these interventions.

In the present study, the first to our knowledge to describe barriers to implementing tobacco control policies in PSH, we partnered with six PSH agencies in the San Francisco Bay Area to conduct a formative assessment of tobacco control interventions and to provide a framework for a toolkit of strategies to reduce tobacco use among PSH residents. This study reports on the perspectives of staff in PSH, while an accompanying manuscript reports on the perspectives of residents (Petersen, Elser, Nguyen, Alizaga, & Vijayaraghavan, 2018).

## METHOD

### Setting and Participants

We asked directors and staff from PSH agencies to complete questionnaires and participate in in-depth, semistructured interviews on attitudes toward and barriers to implementing smoke-free policies and cessation services in PSH. We identified 23 properties within the six agencies that were thought to be the highest priority based on clientele burden of tobacco use. We used a purposive sampling strategy to recruit the directors of each agency (“directors”), staff who provided supportive services (“service staff,” e.g., case managers, nurses, counselors), and staff who offered property management (“management staff,” e.g., property managers) from each of the 23 properties who were 18 years or older and able to provide informed consent. Participants were reimbursed $35 for participating in the study. Using survey items that we previously developed ([Bibr R34][Bibr R34], 2015b), we administered the questionnaire on paper and entered the responses into an online database. We conducted the interviews in a private room at each property after completion of the questionnaire. The University of California, San Francisco institutional review board approved all study procedures.

### Quantitative Measures

#### PSH Characteristics and Smoke-Free Policies.

Directors reported the average length of stay and resident demographics across all properties. Directors reported whether their properties had smoke-free policies indoors in living areas and/or shared areas or in outdoor areas, designated smoking zones, written policies that restricted the use of marijuana or electronic cigarettes indoors, written policies in the lease agreement, and whether their county had a smoke-free ordinance.

#### Attitudes Toward Smoke-Free Policies and Cessation Services.

We asked participants to report their level of agreement with statements on attitudes toward and barriers to implementing smoke-free policies and providing tobacco cessation treatment, and directors and management staff reported barriers to enforcement. We obtained responses using a 5-point Likert-type scale (*strongly agree, agree, neither agree nor disagree, disagree, strongly disagree*).

#### Smoking Behaviors and Demographics.

Participants reported whether they had ever smoked (i.e., smoked at least 100 cigarettes during their lifetime), and ever smokers reported whether they were current smokers (i.e., smoked daily or some days) or former smokers (i.e., not smoking at all at the time of the survey). Current smokers reported their daily cigarette consumption on smoking days, time to first cigarette after waking (within 5 minutes, 6–30 minutes, 31–60 minutes, or after 60 minutes), intention to quit smoking (never expect to quit, may quit in the next 6 months, will quit in the next 6 months, or will quit in the next month), and whether they had attempted to quit within the past year. Participants also self-reported their age, gender, race/ethnicity, and education.

### Qualitative Measures

The social cognitive theory (SCT; Bandura, 1986) served as a framework for the in-depth, semistructured interview guide. The SCT posits that behavior change at the individual or systems level is dependent on the interaction among environmental, personal, and behavioral factors. We asked staff to describe how environmental (e.g., social norms), personal (e.g., attitudes toward smoke-free policies), and behavioral (e.g., personal smoking behavior) factors influenced the adoption of smoke-free policies and implementation of cessation programs. We asked participants to answer questions on their roles, the range of supportive services offered, their perspectives on the culture of tobacco and substance use among residents, their attitudes toward and barriers to implementing smoke-free policies in PSH, their perspectives on maintenance costs attributable to tobacco use, and ideas for implementing smoke-free policies and cessation services in PSH. Using this information and constructs from the SCT such as behavioral capability (knowledge/skills), reinforcements (internal/external reinforcements, social norms), and self-efficacy (Bandura, 1986), we presented a framework for developing a toolkit of strategies to address tobacco use at the individual, organizational, and policy levels. We relied on prior models to address tobacco dependence among smokers with mental illness (Correa-Fernández et al., 2017; Williams et al., 2011) that have integrated clinical treatment, community support, and environmental changes (including smoke-free policies) to help smokers quit.

### Quantitative Data Analysis

For all descriptive statistics, we used means (*SD*) for continuous variables and proportions for categorical variables. We showed differences in demographic and tobacco use characteristics among directors, management staff, and service staff using chisquares and summary statistics. We dichotomized responses to statements on attitudes toward treating tobacco dependence as “strongly agree or agree” versus “strongly disagree, disagree, or neither agree nor disagree.” We calculated the average attitudes score for each participant, with scores ranging from 1 (*least favorable*) to 5 (*most favorable*) and reverse coded negatively worded items. We then presented group-level (e.g., directors, management staff, or service staff) averages for the attitudes and barriers domains. We conducted all analyses using Stata, Version 12.1 (StataCorp, 2011).

### Qualitative Data Analysis

The in-depth, semistructured interviews were audiotaped and transcribed verbatim by a contracted professional transcription service. Personal identification data were redacted. We used ATLAS.ti, Version 8.1.28 (ATLAS.ti Scientific Software Development, 2018) qualitative data analysis software to facilitate coding and a grounded theory approach to analyze the transcripts (Corbin & Strauss, 2015). Two coders coded all transcripts, and the principal investigator reconciled the codes. After developing a preliminary codebook, we iteratively coded the transcripts and resolved disagreements in assignment or description of codes through discussion and consensus. We further refined the number of overall codes by grouping them into a short list of themes and identified linkages among themes to develop a toolkit of strategies for PSH programs to develop tobacco control programs. Cohen’s kappa score for intercoder reliability was 0.70. We used quotations to reflect the themes.

## RESULTS

### No Smoking Policies and Smoking Cessation Services in Supportive Housing Sites in the Bay Area

We obtained responses from 6 directors, 23 management staff, and 24 service staff representing the six agencies (Table 1). Directors reported that 40.9% of their residents identified as Black and 29.9% as White. All except two sites allowed smoking in indoor living areas. The mean age of staff was 51.2 years, and they were racially and ethnically diverse (Table 2). Approximately half (45.3%) were ever smokers, and the quit ratio (ratio of former/ever smoker) was 44.4% for service staff, 50% for management staff, and 100% for directors.

Almost all staff (92.9%) agreed that smoke-free policies are important because they provide a clean, safe environment for staff to work in and clients to live in (Table 3). Most staff (71.6%) agreed that they did not have the appropriate expertise to offer smoking cessation services. Never smokers (*n* = 18, 62.1%) were more likely than former (*n* = 5, 35.7%) or current smokers (*n* = 0, 0%) to agree that smoking contributes to property maintenance costs and that having a no-smoking policy indoors will lead to reduced maintenance costs (*n* = 24, 82.8% vs. *n* = 5, 29.2% vs. *n* = 3, 12.5%).

### Qualitative Themes

We identified two themes from the in-depth, semistructured interviews: the role of PSH in promoting tobacco reduction or cessation and attitudes toward smoke-free policies (Table 4).

#### The Role of PSH in Promoting Tobacco Reduction or Cessation.

All staff believed in the harm reduction model of PSH and reported engaging with clients through active case management to address barriers to maintaining housing. We identified three subthemes: harm reduction principles of PSH, staff perspectives on their roles in encouraging smoking reduction or cessation, and residents’ mental illness and substance use as barriers to cessation.

#### Harm reduction principles of PSH.

Participants emphasized that clients’ housing stability was their primary priority. Staff made the distinction that PSH was “housing” and not a “treatment recovery program”; residents had the same freedoms as people in market rate housing. Some staff reported that substances with the potential for legal complications (e.g., opioids) were more detrimental than tobacco use, and used the substitution of tobacco in lieu of these other substances as a harm reduction strategy. A minority of staff believed that all addictions, including nicotine, were similar, and encouraged residents to reduce all substance use provided that the resident initiated the efforts.

#### Staff perspectives on their roles related to smoking reduction or cessation.

Management staff reported that their primary interactions with residents were through rent collection, addressing lease violations, or property management. Some management staff discussed their use of tobacco with clients to establish a therapeutic alliance. A few management staff supported the use of tobacco to allay residents’ anxiety or stress.

While some service staff were supportive of engaging residents in discussions around tobacco use, a minority reported that restricting smoking indoors or discussing smoking cessation, when residents did not initiate the discussion, contradicted harm reduction. Some staff recognized the role of tobacco use in increasing financial strain and suggested that discussing tobacco use in the context of financial stability was feasible.

Two directors reported that under the federal fair housing criteria, which “prohibits housing discrimination on the basis of race, color, religion, sex, disability, familial status, and national origin,” they were unable to ask residents about their tobacco use because it could be viewed as discriminatory. However, the remaining four directors supported procedures that required staff to screen for tobacco use on entry into housing.

#### Mental illness and substance use as barriers to restricting smoking behavior among residents.

Service and management staff reported instances when residents with severe mental illness and/or substance use disorders were unable to comply with restrictions on indoor smoking in shared areas. Staff anticipated that residents with depression or agoraphobia would find it challenging to adhere to a smoke-free rule that prohibited smoking in indoor living areas and expected that lease violations would increase. In properties that had implemented smoke-free policies, staff reported instances of having to provide written violations leading to the threat of eviction, a consequence that none of the staff believed was ethical.

Several staff reported that tobacco use alleviated symptom severity among residents with severe mental illness, believing that “nicotine helped calm certain mental illnesses.” A director reported that addressing smoking behaviors without acknowledging the role that smoking plays in relieving stress would dilute the benefits of cessation counseling.

#### Attitudes Toward Smoke-Free Policies.

There was no consensus around the feasibility of implementing indoor smoke-free policies in PSH. While some staff reported that smoke-free policies would be difficult to implement due to resident resistance, others stated that residents would eventually accept the policy if provided sufficient education. Some staff believed that policies could compromise housing stability if residents chose to leave the property or received repeated violations that would lead to a threat of eviction. Two subthemes emerged: smoker’s rights, and barriers to implementation and enforcement of smoke-free policies.

#### Smoker’s rights.

A few staff reported that broaching indoor cigarette smoking with residents would violate their smoker’s rights. A service staff participant stated, “This is their home, and if they choose to smoke, then they choose to smoke.” However, another management staff member reported that although tobacco use was an individual’s choice, they could facilitate cessation by offering cessation services.

#### Barriers to implementation and enforcement of smoke-free policies.

While most staff were supportive of policies that restricted smoking in indoor living areas, lack of appropriate enforcement tools was a barrier to implementation. Staff anticipated having to spend a substantial portion of their day monitoring a policy without adequate tools to distinguish cigarette smoke from that of other substances. Although staff could provide warnings or lease violations, in the absence of firm repercussions, the policy would be difficult to enforce. None of the staff believed that eviction for a smoking violation was appropriate or ethical. A management staff member stated that having a local ordinance to implement smoke-free policies would provide the impetus to enforce such a policy.

In buildings that had a smoke-free policy in indoor living areas, staff reported that residents could evade detection by smoking next to a window. While staff felt that the receipt of a lease violation and/or threats of eviction were enough of a deterrent, they expressed interest in finding ways to enforce policies without having to rely on punitive measures.

Staff suggested that accommodations would be necessary to help some residents comply with an indoor smoke-free policy in living areas, including better signage to remind residents with cognitive impairments or housing residents with disabilities on the first floor to facilitate smoking outside.

### Framework to Develop a Toolkit of Strategies to Implement Tobacco Control Interventions in PSH

Using these formative assessments, we created a framework to develop a toolkit of strategies that encompassed policy, organizational, and individually tailored targets to address tobacco use among PSH clientele (Supplemental Table S1, available in the online version of this article).

At the policy level, we recommend the adoption of voluntary smoke-free homes as a pathway to a smokefree building and as the first step to increasing access to smoke-free housing for formerly homeless adults. To increase resident self-efficacy in adopting home smoking restrictions, service staff would benefit from training on how to counsel residents on adopting a smoke-free home. Such policies could be disseminated with cityand county-level support through smoke-free ordinances in multiunit housing.

At the organizational level, to increase support for smoke-free policies, such policies must be accompanied with services for cessation. Insofar as supportive housing is a one-stop for most services, supportive housing staff can be incentivized to receive training on how to incorporate tobacco use screening during intake assessments, provide brief cessation counseling, and integrate social services with medical services so that recommendations for cessation medications are available on-site. Partnerships with local health care and tobacco control organizations can increase capacity to provide on-site cessation services, thereby disseminating cessation services widely across PSH sites.

For individually targeted interventions, services staff in supportive housing can be trained to use discussions around rent evasion, substance use, food insecurity, or financial hardship to address tobacco use and to frame tobacco cessation as an intervention that will not only improve health but also mitigate financial burden. Case management discussions can also highlight improvements in mental health symptoms and the potential to reduce co-use of other substances after smoking cessation.

## DISCUSSION

In this study of staff in PSH for formerly homeless adults, the majority of staff agreed that smoke-free policies were important and would support further changes to protect nonsmokers from secondhand smoke. However, barriers included concerns that policies contradicted the harm reduction framework of PSH, the lack of adequate smoking cessation resources for residents, and the lack of tools to enforce a smokefree policy. Findings from our study highlight potential policy, organizational, and individually tailored strategies to address these barriers.

Harm reduction is one of the primary tenets of PSH. Restricting indoor smoking posed a theoretical and practical challenge because it appeared to interfere with freedoms afforded to residents. In particular, restricting smoking when not restricting other substance use posed a conflict. However, staff recognized that unlike other substance use, continued smoking and exposure to secondhand smoke increased harm to nonsmoking residents and staff.

Research from correctional facilities (Kennedy, Davis, & Thorne, 2015), inpatient psychiatric facilities (Hehir, Indig, Prosser, & Archer, 2013), and multiunit housing (Cramer, Roberts, & Stevens, 2011; King, Travers, Cummings, Mahoney, & Hyland, 2010) has shown that staff dissent on implementing smoke-free policies was common in the early stages of implementation. Arguments against the implementation of smoke-free policies in correctional facilities and inpatient psychiatric facilities stemmed from misconceptions that client dissent would increase (Kennedy et al., 2015; Lawn & Pols, 2005; Prochaska, Gill, & Hall, 2004; Shmueli, Fletcher, Hall, Hall, & Prochaska, 2008). However, these concerns were unfounded. Barriers to implementation in multiunit housing included concerns about enforcement, tenant objections, rapid turnover, increase in vacancy, and loss of market share (Cramer et al., 2011).

Prior studies have suggested that if policies were implemented in a manner that allowed for client and staff agreement, the likelihood of policy success was greater (Kennedy et al., 2015; Lawn & Pols, 2005). Other factors associated with successful implementation included providing a forum for staff and clients to offer feedback on the implementation and enforcement process and linking policy implementation with a clear pathway to cessation treatment (Pizacani, Maher, Rohde, Drach, & Stark, 2012). In this study, staff in sites that had enacted smoke-free policies in indoor living areas reported making reasonable accommodations such as housing individuals with disabilities in units next to exits to facilitate smoking outside and providing smoking cessation resources to increase compliance with the policy.

One of the first PSH sites to implement smoke-free policies showed that support for an indoor smoke-free policy was highest among current smokers (Petersen et al., 2018). Staff did not perceive there to be a difference in the frequency of complaints between smokers and nonsmokers, and there were no evictions related to the policy. Voluntary adoption of smoke-free homes is another potential pathway to a smoke-free building and is a powerful indicator of community social norms around smoking (Vijayaraghavan et al., 2013).

The recent implementation of the smoke-free policy rule in public housing authority housing in the United States could present new opportunities for PSH (Department of Housing and Urban Development, 2016). In public housing for low-income residents, smokefree policies have been shown to reduce cigarette

consumption, increase quit rates, and decrease indoor secondhand smoke exposure; property management also report incentives such as reduced liability and costs (King et al., 2010; Pizacani et al., 2012). Although public housing authority housing is distinct from PSH in clientele and regulatory authorities, their experiences could inform implementation strategies.

Many homeless adults use cigarettes to cope with the stressors of homelessness (Vijayaraghavan et al., 2018; Vijayaraghavan, Hurst, & Pierce, 2017); thus, understanding the role that nicotine dependence plays in these individuals’ lives is crucial to implementing policies to restrict smoking. The co-occurrence of mental illness and substance use disorders poses significant barriers to smoking cessation and adherence to a smoke-free policy (Vijayaraghavan et al., 2017; Vijayaraghavan et al., 2018). Cessation interventions that are integrated with substance use treatment may offer an effective modality to introduce cessation services in PSH (Williams et al., 2011). Pharmaceutical aids increase abstinence duration when accompanied with smoke-free home policies (Gilpin et al., 2006). Thus, minimizing barriers to access medications may mitigate withdrawal symptoms that smokers might experience when faced with smoking restrictions. Behavioral health care professionals may be unprepared to counsel smokers on tobacco cessation despite believing that it is their responsibility to do so (CorreaFernández et al., 2017; Williams et al., 2011). As such, training PSH staff to offer smoking cessation counseling and ensuring access to smoking cessation aids could increase adherence to a smoke-free policy while helping residents quit smoking.

The primary goal of PSH is to ensure housing stability. Previous studies have shown that cigarette smoking can contribute to up to a third of monthly expenditures, representing a substantial financial burden in a population with limited resources (Baggett et al., 2016; Wrighting, Businelle, Kendzor, LeBlanc, & Reitzel, 2017). Staff reported that while they acknowledged the financial burden of smoking among clientele, no staff addressed smoking in the context of financial management. Our study highlights an opportunity to introduce discussions around smoking cessation in the context of financial burden and the benefits of smoking cessation in increasing financial capital.

## Limitations

Our study had several limitations. We used a purposive sampling strategy to recruit staff; therefore, perspectives may not reflect those of peers in the same facility or others in the United States. Given that California has a robust clean indoor air policy and has enacted several policies to encourage tobacco cessation (e.g., increased tobacco tax), staff attitudes may be different in other states where tobacco control has been less of a priority. The PSH agencies that did not participate in this study may differ in important ways, and therefore there may be a potential selection bias. However, strengths of the study included the use of quantitative and qualitative methods, the focus on perspectives from services and property management staff in PSH, and our partnership with six agencies throughout the San Francisco Bay Area that provide housing in total to over 4,000 formerly homeless residents.

## Conclusions

Providing a supportive environment to increase smoking cessation by implementing smoke-free policies and offering cessation services will reduce harm from tobacco use and may also provide a pathway to housing stability by increasing disposable income. To support the implementation of indoor smoke-free policies in PSH, it is crucial that they are implemented in conjunction with cessation care that frames tobacco cessation in the context of improving mental health and substance use outcomes and reducing financial strain.

## Supplementary Material

Supplemental Table S1

## Figures and Tables

**TABLE 1:** Supportive Housing Site Characteristics and Smoke-Free Policies

Characteristic/Policy	Agency 1	Agency 2	Agency 3	Agency 4	Agency 5	Agency 6
Agency characteristics						
Total no. of residents housed^a^	1,353	450	700	125	500	619
Length of stay in years, *M*	5	4	7	10	5	5
Tenant characteristics^b,c^						
% Male	53	77	68	85	98.5	49.4
% Non-Hispanic Black	39	31.5	75	28	45	27
% Non-Hispanic White	24	35.2	20	28	45	41
% Hispanic/Latino	19	16	1	28	15	27
% Asian/Pacific Islander/Native American/multiracial/other race	27	12.3	5	13	6	17
Smoke-free policies						
Smoking allowed in individual living areas	Yes	Yes	Yes	No	No	Yes
Smoking allowed on patios, balconies, and porches	Yes	Yes	Yes	No	Yes	No
Designated outdoor smoking zones	No	No	No	Yes	Yes	No
Policies restrict marijuana indoors	Yes	No	Yes	Yes	Yes	Yes
Policies restrict e-cigarettes indoors	No	No	No	Yes	No	Yes
Written policies in the lease agreement	Yes	Yes	No	Yes	Yes	Yes
County smoke-free ordinance	No	No	Yes	Yes	No	Yes

a Total number of residents across all their properties.

b Percentages may not equal 100% since participants were allowed to select more than one category.

c Percentage of transgender residents ranged from 0% to 3% across all sites.

**TABLE 2:** Staff Smoking Behaviors and Demographics

*Demographics/Behaviors*	*Agency Director,*n = *6*	*Individual Site Property Manager,* n = *23*	*Supportive Services Staff,* n = *24*	*All,* N = *53*
Age, *M (SD),* years	51.2 (7.8)	48.8 (10.2)	41.5 (14.2)	45.8 (12.5)
Female, *n* (%)^a^	5 (83.3)	16 (69.6)	12 (50.0)	33 (62.3)
Race/ethnicity, *n* (%)				
Non-Hispanic White	6 (100)	6 (26.1)	7 (29.2)	19 (35.8)
Non-Hispanic Black	0 (0)	10 (43.5)	9 (37.5)	19 (35.8)
Hispanic/Latino	0 (0)	4 (17.4)	4(16.7)	8 (15.1)
Asian/Pacific Islander/Native American/multiracial/other race	0 (0)	6 (26.0)	5 (20.9)	15 (28.3)
Education, *n* (%)				
High school or general equivalency diploma	0 (0)	1 (4.3)	0 (0)	1 (1.9)
Some college	0 (0)	9 (39.1)	6 (25)	15 (28.3)
College and/or other professional training	6 (100)	13 (56.5)	18 (75)	37 (69.8)
Smoking characteristics, *n* (%)				
Ever smoker^b^	3 (50)	12 (52.2)	9 (37.5)	24 (45.3)
Former smoker^c^				
Quit in the past year	0 (0)	2 (8.7)	0 (0)	2 (3.8)
Quit more than a year ago	3 (50)	4 (17.4)	5 (20.8)	12 (22.6)
Current smoker^d^	0 (0)	6 (26.1)	4 (16.7)	10 (18.9)
Smoking behaviors among current smokers				
Average daily cigarette consumption, *M* (*SD*)	0 (0)	12.5 (4.6)	6 (3.5)	10.3 (5.2)
Time to first cigarette after waking < 30 minutes, *n* (%)	0 (0)	2 (33.3)	3 (75.0)	5 (50.0)
Intention to quit at baseline, *n* (%)				
Never expect to quit	—	1 (16.7)	0 (0)	1 (10)
May quit in the near 6 months	—	0 (0)	4 (100)	4 (40)
Will quit in the next 6 months	—	4 (66.7)	0 (0)	4 (40)
Will quit in the next month	—	1 (16.7)	0 (0)	1 (10)
Quit attempt in the past year, *n* (%)	—	4 (66.7)	2 (50)	6 (60)

a Percentage of transgender staff ranged from 0% to 1.9%.

b Those who reported smoking at least 100 cigarettes in their lifetime.

c Those who reported smoking not at all.

d Those who reported smoking some days or every day.

**TABLE 3:** **Proportion Who Strongly Agreed/Agreed With Statements About Smoke-Free Policies and Cessation Services and Barriers to Treating Tobacco Dependence (*N*** = **53)**

Statement	*Directors,* n = *6*	*Management Staff,* n = *23*	*Service Staff,* n = *24*
Attitudes, *n* (%)			
Smoke-free policies are important because they provide a clean and safe environment for our staff to work in and clients to live in.	6 (100)	21 (91.3)	21 (87.5)
Smoke-free policies may help our tenants and staff quit smoking.	5 (83.3)	14 (60.9)	14 (58.3)
I would prefer if more of my staff are nonsmokers.^a^	6 (100)	11 (47.8)	10 (41.7)
I would support further changes to our smoking policy to protect nonsmokers from secondhand smoke.	5 (83.3)	22 (95.7)	21 (87.5)
If we have a strict smoke-free policy, it will reduce our occupancy rate.	1 (16.7)	6 (26.1)	6(25)
Support to help people quit smoking should be part of the care that we provide.	6 (100)	21 (91.3)	21 (87.5)
We have adequate organizational support and resources to offer smoking cessation support to our clients.	3 (50.0)	4 (17.4)	9 (37.5)
Smoking cessation is not a feasible goal for our clients.	1 (16.7)	3 (13.0)	4 (16.7)
Because smoking is a personal choice, it is up to our tenants whether they smoke or not; we should not interfere one way or the other.	2 (33.3)	5 (21.7)	6 (25.0)
Smoking contributes significantly to our property maintenance costs.	4 (66.7)	11 (47.8)	8 (33.3)
I think that having a no-smoking policy indoors will lead to reduced maintenance costs.	4 (66.7)	13 (56.5)	15 (62.5)
Average score for attitude items, *M (SD)*^b^	3.92 (0.40)	3.69 (0.38)	3.60 (0.44)
Barriers, *n* (%)			
Monetary constraints make it hard for us to offer smoking cessation services.	2 (33.3)	7 (30.4)	10 (41.7)
Constraints on staff time make it hard for us to train staff to offer smoking cessation counseling.	4 (66.7)	11 (47.8)	13 (54.2)
We do not have the appropriate expertise to offer smoking cessation services to our clients.	5 (83.3)	14 (60.9)	17 (70.8)
Tenants’ other priorities make smoking cessation less of a priority.	4 (66.7)	15 (65.2)	21 (87.5)
Tenants smoking indoors/not following policy.^c^	3 (50.0)	12 (52.2)	—
Lack of time to enforce the policy.^c^	2 (33.3)	3 (13.0)	—
Lack of resources to support smoking cessation for tenants and staff.^c^	2 (33.3)	13 (56.5)	—
Concern for tenants’ rights to smoke.^c^	3 (50.0)	6 (26.1)	—
Concern for occupancy rates.^c^	1 (16.7)	3 (13.0)	—
Tenants smoking too close to the no-smoking areas.^c^	3 (50.0)	15 (65.2)	—
Average score for barrier items, *M( SD)*^b^	2.68 (0.55)	2.73 (0.35)	2.43 (0.83)^d^

a *p* = .02.

b Average scores represent a group-level average for each domain. A higher score is indicative of a. more favorable attitude or a greater number of barriers toward treating tobacco dependence.

c Service staff did not respond to these barriers items.

d Score is based on items where all three staff groups responded.

**Table 4:** Permanent Supportive Housing Staff Quotes From In-Depth, Semistructured Qualitative Interviews

	The role of supportive housing in promoting tobacco reduction or cessation
Harm reduction principles of permanent supportive housing	Since one of our major goals is to keep people housed and prevent re-entry into homelessness . . . we’ll talk to them about how their behaviors that stem from their substance use are putting them at risk of becoming homeless, or putting them at risk of law enforcement involvement, or putting them at risk of major health problems . . . and really try to help them to figure out how do they mediate their use, so that their behaviors can get more in line with what they want. (Agency director, Agency 3)
	It’s an SRO [single-room occupancy], it’s not a treatment program . . . we’re not really trying to help them become sober from certain substances—whether that be tobacco, alcohol or the harsher drugs, unless they ask for our support in becoming sober. (Supportive services staff, Agency 2)
Staff perspectives on their roles in encouraging smoking reduction or cessation	It calms them and changes them, and so stepping outside and smoking a cigarette on the sidewalk, or going into their room and staying there all day, it’s almost easier for staff. (Property manager, Agency 6)
	We wouldn’t even ask them about their substance use, or the other thing might be in the room, we want to try to work around that, that comes out in the motivating interviewing—we worked with what they call a harm reduction model, so basically, we know the client, the tenant is the driver of the car and we ride in the back and we let them decide what they want to do. (Supportive services staff, Agency 1)
Mental illness and substance use as barriers to cessation	There’s a lot of folks that are struggling with depression, they don’t want to be social, they remain pretty isolated and like to have the safety of this room ... to go outside and have to interact with the world. . . . Maybe they don’t really want that, especially for people that smoke a pack a day, imagine, twenty times going up and down and having to interact with the world, is a lot. . . . And I could see property management writing a ton of lease violations, and that being—the idea that people would get evicted for smoking in their rooms sounds a little extreme. (Supportive services staff, Agency 2)
	I think tobacco is the most pernicious substance that my tenants use, and they use everything. . . . And yet, in 2009,1 saw this guy half-naked, who had Stage 4 lung cancer, walking to a store to get cigarettes. So what do you offer that guy? (Agency director, Agency 2)
	Attitudes toward smoke-free policies
Smoker’s rights	There are individuals that refuse to quit because they believe it’s their right, their personal choice, and you can’t stop me. And I somewhat agree, because everybody has rights, and you can’t—just because I don’t like smoking, I can’t tell the person to quit. So we know that second-hand smoke is bad for you, but I can’t—just because I don’t like smoking, I can’t tell the person to stop, because they have rights, too. (Property manager, Agency 6)
	The thing that I see is that when you realize, when you factor in how much money I’m spending on cigarettes, what is it else that I could do with that money. . . . Sometimes those who smoke don’t often see what a huge portion of their limited income cigarettes are, and so also, I think what other things they would do with that money. (Agency director, Agency 5)
Barriers to implementation and enforcement of smoke-free policies	Our job is to house people, and if we have an issue with a person smoking, we basically, legally, we have to issue them a lease violation and subsequently, there will be multiple lease violations, but it’s very difficult to actually go to court and evict somebody, just because someone smokes. (Property manager, Agency 6)
	And the enforcement is challenging, because number one, it’s very difficult to tell where smoke is coming from in particular, and then because it’s permanent supportive housing, we treat them just like anybody else who rented an apartment—you can’t just barge into the unit and see evidence. You have to give 24-hour notice, and of course by then it’s very difficult to see signs of smoking, and of course, people deny it, and it’s just very challenging. And the courts will not support an eviction for smoking. (Agency director, Agency 5)
