# Supplementary material for: Developing Tobacco Control Interventions in Permanent Supportive Housing for Formerly Homeless Adults
Source: Health Promot Pract. Author manuscript; Available in PMC 2020 Oct 11. (PMC6788936; doi:10.1177/1524839919839358)
Supplement: Supplemental Table S1 [file NIHMS1023962-supplement-Supplemental_Table_S1.pdf]

Table 5. Toolkit strategies

Barrier identified	Potential toolkit strategies	Dissemination effort
Feasibility of implementing smoke-free policies in permanent supportive housing	• Encourage voluntary adoption of smoke-free homes<br>• Train staff on how to counsel residents on adoption a smoke-free home<br>• Apply lessons learned from sites that have implemented smoke-free policies<br>• Solicit resident and staff feedback to identify repercussions for violations	• Designate a smoking zone; make reasonable accommodations for individuals with disabilities<br>• Work with city/county officials to get leadership support for implementing smoke-free policies in supportive housing
Barriers to cessation services and delivery	• Incorporate tobacco use screening and brief counseling upon entry into housing<br>• Integrate medical and social services so recommendations for nicotine replacement therapies (NRT) are available for those who want it<br>• Train staff to screen and counsel for tobacco use	• Partner with local health care organizations and tobacco control organizations to offer technical assistance and/or capacity building to provide cessation services (e.g., training staff, developing scripts for smoking cessation counseling, linking with health care systems) to supportive housing<br>• Work with substance use programs to advocate for comprehensive treatment of all addictions and the provision of integrated treatment of tobacco dependence
Co-use of tobacco and other substances	• Integrate discussions around substance use and mental illness with nicotine dependence<br>• Discuss substance and tobacco use in the context of financial strain	• Train staff to counsel residents on how co-use may threaten housing or financial stability, highlighting potential benefits of smoking cessation including improvements in mental health symptoms and substance use outcomes
